# Effects of 12-month physical and cognitive training on sarcopenia determinants in older adults: a subgroup analysis of a randomised clinical trial

**DOI:** 10.1007/s40520-025-02935-7

**Published:** 2025-02-06

**Authors:** Onni Oskari Hämäläinen, Tiina Marketta Savikangas, Anna-Katriina Tirkkonen, Markku Juhani Alén, Arto Jorma Hautala, Sarianna Sipilä

**Affiliations:** 1 Faculty of Sport and Health Science, University of Jyväskylä, Jyväskylä, Finland; 2 Gerontology Research Center, University of Jyväskylä, Jyväskylä, Finland; 3 Department of Medical Rehabilitation, Oulu University Hospital, Oulu, Finland

**Keywords:** Resistance training, Walking training, Cognitive training, Sarcopenia, Physical activity, Muscle strength, Muscle mass

## Abstract

**Background:**

Low physical activity is a major risk for sarcopenia. Whether training according to physical activity guidelines accompanied with cognitive training is effective on sarcopenia, remains unclear.

**Aims:**

We investigated whether the effects of 12-month physical and cognitive training (PTCT) and physical training (PT) on grip and knee extension strength, muscle mass, and walking speed differed between older adults with and without sarcopenia.

**Methods:**

Community-dwelling older adults (*N* = 314, mean age 74.5 ± 3.8 years, 60% women) who did not meet physical activity guidelines were randomized to PTCT and PT groups. PT for both groups included supervised and home-based multicomponent physical training. Cognitive training (CT) included computer-based exercises for executive functioning. Sarcopenia was determined according to the European Working Group on Sarcopenia in Older People 2019 criteria. Generalized estimation equation analysis were conducted.

**Results:**

Compared to PT, PTCT had no additive effect on strength, muscle mass, or walking speed in participants with or without sarcopenia. In pooled data (PT + PTCT) change in the grip strength was greater in sarcopenia (*n* = 49) group compared to non-sarcopenia (*n* = 264) group (interaction, *p* =.014). Both groups improved knee extension strength, and walking speed, but no statistically significant difference between the groups were observed. Muscle mass did not change in either group.

**Conclusion:**

Physical training according to physical activity recommendations improves muscle strength, walking speed, and maintains muscle mass in sarcopenia. Additional cognitive training had no benefits on these outcomes.

**Trial registration number:**

ISRCTN52388040 and date of registration 20/1/2017.

**Supplementary Information:**

The online version contains supplementary material available at 10.1007/s40520-025-02935-7.

## Introduction

Sarcopenia is an important condition compromising healthy ageing and physical performance in older adults and it is characterised by accelerated loss of muscle strength, muscle mass, and in addition, poor physical function [1]. The European Working Group on Sarcopenia in Older People (EWGSOP2) has proposed different stages of sarcopenia, probable sarcopenia, defined as low muscle strength, and confirmed sarcopenia defined as low muscle strength and muscle mass. In addition, sarcopenia can be classified as severe if walking speed is low [2]. The main reasons for developing sarcopenia are age-related lifestyle factors such as decreased levels of physical activity and poor nutrition, various diseases, chronic inflammation and hormonal changes, all of which accelerate muscle loss [3]. The global prevalence of sarcopenia in adults over 60 years varies from 10 to 27% depending on the sarcopenia classification used [4], and the prevalence of sarcopenia is predicted to increase as the population ages [5].

Sarcopenia is associated with a variety of adverse outcomes, including falls, hospitalisation, all-cause mortality [6], and increased healthcare costs [7]. In terms of treatment and recovery in different patient groups, sarcopenia is associated with longer hospital stays, severe complications, postoperative infections and higher mortality compared to those without sarcopenia [8]. Sarcopenia is an important health issue and therefore acceptable and safe prevention and treatment strategies for sarcopenia are needed, with a focus on older people.

To counteract and manage sarcopenia, resistance training has been shown to be most effective in increasing muscle strength and functional capacity and maintaining muscle mass in older adults with sarcopenia [9, 10]. In addition, higher levels of physical activity, and in particularly moderate-to-vigorous physical activity, have been shown to be inversely associated with sarcopenia in both cross-sectional and longitudinal settings [11]. Therefore, interventions that include both resistance training and physical activities according to the physical activity recommendations could be effective in the prevention and treatment of sarcopenia.

In addition to physical training, cognitive training has been proposed as a possible intervention to improve physical function and walking in older people [12]. As successful mobility in every day environment requires different cognitive processes, such as executive functioning [13], it may be hypothesized that cognitive training with focus on executive functioning effects on physical functions as well. In addition, due to overlapping neural networks and brain areas that are associated with both executive functions and dual-task walking speed may provide a mechanism by which cognitive training can improve physical function [14]. In a recent meta-analysis, cognitive training in addition to physical training had a small effect on overall physical function, but no effects on physical sub-domains, such as strength or walking speed in older adults with mild or no cognitive impairment [15]. To the best of our knowledge, there are no studies exploring the effects of combination of physical and cognitive training on sarcopenia, and in our study, we included a cognitive component as an experimental approach to treating sarcopenia.

Those with relatively lower strength or muscle mass levels may have greater absolute adaptations to resistance training in strength and muscle mass, when compared to those with greater strength levels at the baseline [16]. However, due to aging and age-related inflammation and physical inactivity, the anabolic resistance to resistance training and, for example, ingested protein may have increased [3]. Thus, we hypothesize that older adults with sarcopenia have greater improvements in strength and muscle mass, whereas those with severe sarcopenia may have reduced strength and mass adaptations when comparing to those without sarcopenia.

Therefore, this study investigated whether the effects of 12-month physical training including the components of American College of Sports Medicine (ACSM) 2007 physical activity guidelines [17] and additional cognitive training on muscle strength on muscle mass and habitual walking speed differed between sarcopenia and non-sarcopenia groups in older adults who did not meet physical activity guidelines prior to the intervention.

## Materials and methods

This study is a subgroup analysis of the single-blinded, parallel-group randomized controlled Promoting Safe Walking among Older Adults (The PASSWORD) trial [18]. The PASSWORD -study was conducted at the University of Jyväskylä, Finland. The primary objective of the PASSWORD- study was to investigate the effects of 12-month physical and cognitive training (PTCT) on maximal walking speed and executive functions compared to physical training (PT) alone among community-dwelling older adults who did not meet physical activity recommendations.

### Participants

**Fig. 1:**
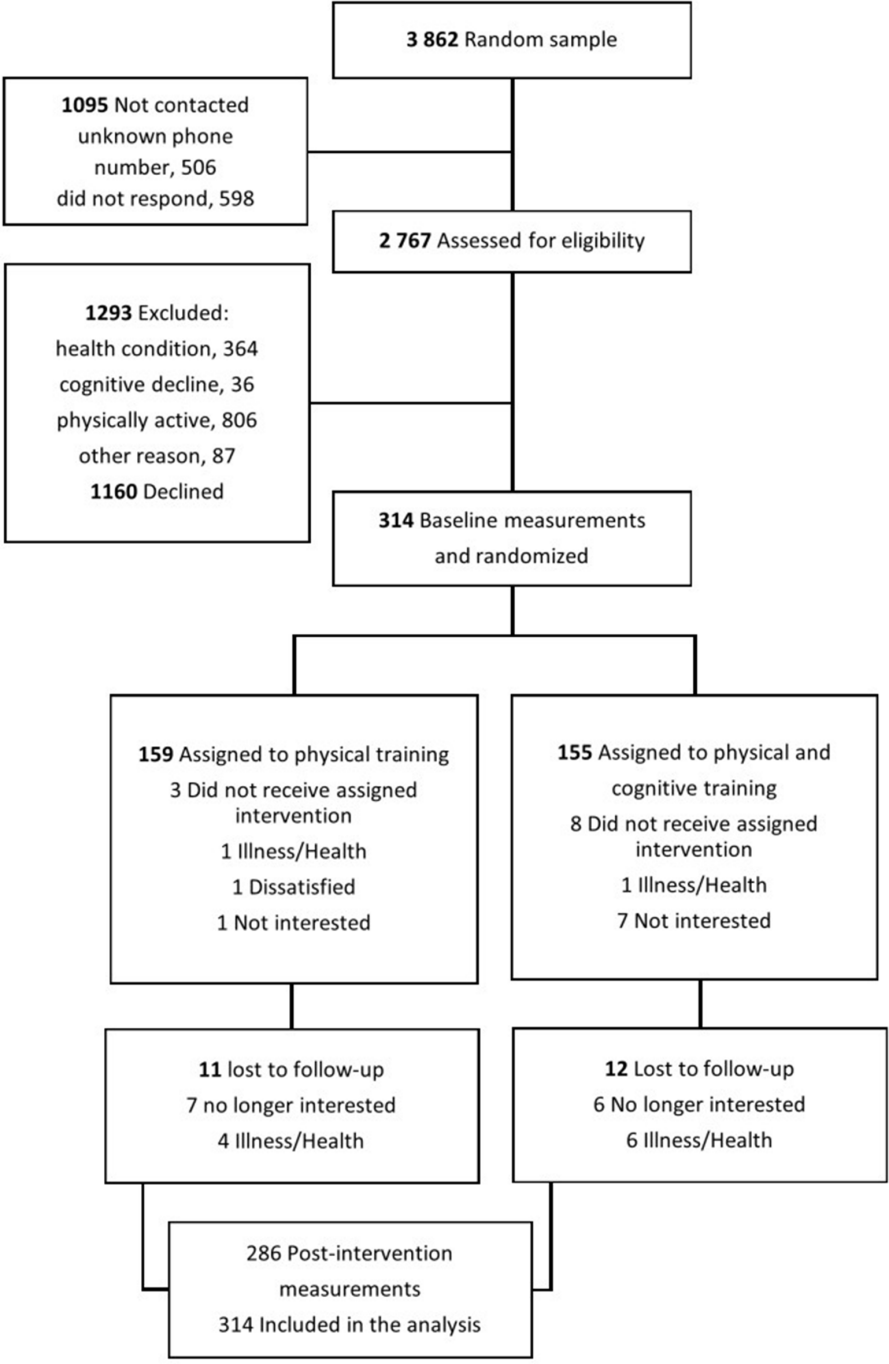
Flow chart of the PASSWORD study

An initial random sample of 3862 people living in Central Finland region was drawn from Finland’s Population Information System administered by the Population Register Center and a total of 314 older adults (60% women), mean age 74.5 ± 3.8 years, were recruited for the study (Fig. 1). Participants were 70- to 85-year-old community-dwelling older adults, who did not meet the physical activity guidelines at the time (had less than 150 min of moderate physical activity in bouts of 10 min per week and no regular resistance training during the past year) [17]. Other inclusion criteria were The Mini-Mental State Examination score ≥ 24, the ability to walk 500 m without assistance (cane was allowed) and informed consent to participation. The exclusion criteria were severe chronic condition or disease, cardiovascular disease, pulmonary disease, progressive disease (i.e. neoplasm, ALS), para- or tetraplegic, or severe depression that affects cognitive and/or physical function of safety participation in the intervention, excessive alcohol consumption, communication difficulties (i.e. having severe hearing or vision impairment), or if another person from the same household was participating in the PASSWORD -study.

### Physical and cognitive training

The detailed overview of the PASSWORD -study training protocols has been published previously [18]. Participants were randomized in a 1:1 ratio to either PT or PTCT group after the baseline assessment. Physical training for both groups included supervised resistance (including balance) and supervised walking (including balance) training sessions once a week, home exercises with a resistance band 2–3 times per week, and advice to do at least 150 min of moderate physical activity per week in bouts of at least 10 min according to the ACSM physical activity guidelines [17]. Supervised resistance training sessions included both hypertrophy and power training and it was organized in a senior gym equipped with machines utilizing air pressure technology and Smart Card/Smart Touch Software (http://hur.fi/en). Resistance of hypertrophy and power training increased progressively up to 70–80% and 60% of 1 repetition maximum, respectively. Walking sessions were organized on a 400-meter outdoor circular track and, in winter, on a 200-meter indoor oval track.

Computer-based cognitive training in the PTCT group targeted executive functions, more specifically set-shifting, inhibition, and working memory updating. In-house developed computer program (*i*PASS) was used, and participants were advised to do cognitive training 3–4 times a week. One training session lasted for 15–25 min, depending on individual’s skills and performance.

Training adherence was tracked separately for each exercise modality using training logs and diaries. Adherence to the supervised strength and walking training sessions was calculated as the percentage of planned sessions attended by participants. The mean weekly number of home exercises completed, and mean walking minutes were obtained from the monthly physical activity diaries that participants returned during the study. Adherence to cognitive training was based on information from the *i*PASS programme login details.

### Muscle strength, muscle mass and walking speed

All the measurements were done at baseline (before randomization) and after 12-month intervention. Maximal isometric grip and knee extension strength were measured from the side of the dominant hand using a dynamometer chair (Metitur, Palokka, Finland). In both strength measurements, after 2–3 practice trials, participants were encouraged to squeeze the handle or extend the knee as forcefully as possible. In both measurements best performance from three to five maximal attempts was taken as the result. The appendicular skeletal muscle mass index (ASMI, kg/m^2^) was calculated by dividing appendicular skeletal muscle mass assessed with dual-x-ray absorptiometry (DXA, Lunar Prodigy, GE Healthcare, Madison, WI, USA) by the square of the body height. Photocells were used to measure the time to complete the 20 m walk at habitual speed and walking speed (m/s) was calculated [19].

### Sarcopenia

Sarcopenia was assessed using the European Working Group on Sarcopenia in Older People diagnostic criteria [2]. Probable sarcopenia was determined if grip strength was below the cut-off values (< 16 kg for women and < 27 kg for men). Sarcopenia was confirmed if the participant, in addition to low grip strength, also had a low ASMI (< 5.5 kg/m^2^ for women and < 7.0 kg/m^2^ for men). Sarcopenia was considered severe if, in addition to low grip strength and low ASMI, the habitual walking speed was less than 0.8 m/s. Grip strength of one participant was missing and this case was left out from the further analysis. In this study none of the participants had severe sarcopenia. For further analysis probable and confirmed sarcopenia cases were recategorized into a single sarcopenia group.

### Background characteristics

Body anthropometrics were measured using standard procedures. Fat percent was assessed by DXA. Self-rated health status, marital status, education, and smoking status were assessed by questionnaire at baseline. Chronic diseases were checked from healthy registry by study physician. Measurements of all variables have been described previously in detail [18].

### Statistical analysis

Participants were categorized into sarcopenia and non-sarcopenia groups. Descriptive statistics for the sarcopenia and non-sarcopenia groups are presented as means and standard deviations (SD). Normality was examined using Shapiro-Wilk test. Differences in baseline characteristics and training adherence between the sarcopenia and non-sarcopenia groups were assessed using independent samples t-test for normally distributed continuous variables, Mann-Whitey U-test for non-normal continuous variables (training adherences, number of home-based exercises, and walking minutes), and chi-square tests for categorical variables (Fisher’s exact for variables with 2 categories and Pearson chi-square for variables more than 2 categories).

First, separately for both sarcopenia and non-sarcopenia groups linear generalized estimation equation (GEE) models were used to analyse if combination of physical and cognitive training had additional effects on strength, muscle mass, or walking speed compared to physical training alone by exploring if there was time by group interaction between PTCT and PT groups. Models were adjusted with age, sex, resistance training and walking training adherence. As there were no differences in the change in muscle strength, mass, or walking speed between PTCT and PT groups in either sarcopenia group (see supplementary file), PT and PTCT groups were pooled for further analysis.

For the main analyses of the pooled data, linear GEE models adjusted for age, sex, and adherence to supervised resistance and walking training were used to explore study group, time, and time by study group interaction effects on the main outcomes. In addition, an age and sex adjusted GEE model was used to examine the interaction of time by training adherence on the main outcomes separately for resistance training and walking training separately for sarcopenia and non-sarcopenia groups. Cohen’s d for effect sizes were calculated (small effect size = 0.2, medium = 0.5, large = 0.8). Statistical analyses were performed using IBM SPSS Statistics for Windows (Version 28.0, Armonk, NY, USA) and significance level was set at 0.05.

## Results

A total of 314 participants (60% women, mean age 74.5 ± 3.8 years) participated in the study and 286 participants (91%) participated in the post-intervention measurements. There were 28 and 21 participants with sarcopenia in the PTCT (*n* = 155) and PT (*n* = 159) groups, respectively. The sarcopenia and non-sarcopenia groups in the PTCT and PT groups improved in grip strength, knee extension strength, and walking speed, and there were no statistically significant differences between the PTCT and PT groups. No changes in ASMI were observed in any group (see supplementary file).

In the pooled data, baseline characteristics and training adherence for both the sarcopenia and non-sarcopenia groups and differences between groups are shown in Table 1. Participants in sarcopenia group (*n* = 49) were slightly older and had higher fat percent. In all participants, adherence to supervised resistance and walking training was 72 ± 24% and 59 ± 31%, respectively. Participants performed home-based exercise on average 1.4 ± 0.9 times per week and the amount of walking was on average 195 ± 145 min per week. Training adherence was similar between the sarcopenia and non-sarcopenia groups. Baseline values for sarcopenia determinants for the sarcopenia and non-sarcopenia groups are shown in Table 2.

**Table 1:** Baseline characteristics and training adherence of non-sarcopenia and sarcopenia groups and differences between groups

	Non-sarcopenia (*n* = 264)	Sarcopenia (*n* = 49)	*p* value
Age, mean (SD), years	74.2 (3.7)	75.9 (4.1)	**0.006**
Women no. (%)	154 (58)	34 (70)	0.147
Height, mean (SD), m	166.6 (8.8)	162.3 (7.8)	**0.004**
Weight, mean (SD), kg	77.4 (14.1)	74.0 (14.5)	0.123
Body mass index, mean (SD), kg/m^2^	27.9 (4.6)	28.1 (5.4)	0.802
Fat percent, mean (SD), %	35.8 (8.2)	38.3 (8.5)	**0.049**
Marital Status, no. (%)			0.075
Married or living together	173 (66)	25 (51)	
Not married or living with a partner	91 (34)	24 (49)	
Highest education, no. (%)			0.082
Low	41 (16)	7 (14)	
Medium	167 (63)	32 (65)	
High	56 (21)	10 (20)	
Smoking status, no. (%)			0.606
Never smoker	159 (60)	32 (65)	
Former smoker	92 (35)	16 (33)	
Current smoker	13 (5)	1 (2)	
Current self-rated health, no. (%)			0.085
Very good/good	124 (47)	16 (33)	
Average/poor	140 (53)	33 (67)	
Comorbidities			
Multimorbidity (≥ 2 chronic diseases)	183 (69)	37 (76)	0.496
Musculoskeletal pain/disease	30 (11)	8 (16)	0.342
Cardiovascular disease	56 (21)	12 (22)	0.577
Pulmonary disease	30 (11)	9 (18)	0.236
Osteoarthritis	58 (22)	14 (29)	0.355
Diabetes	34 (13)	4 (8)	0.477
Stroke/Transcient ischemic attack	15 (6)	2 (4)	1.00
Heart failure/valve disease	15 (6)	1 (2)	0.482
Osteoporosis	3 (1)	3 (6)	0.051
Adherence to strength training sessions, mean (SD), %	74.0 ± (21.9)	70.8 ± (24.0)	0.394*
Adherence to walking training sessions, mean (SD), %	60.9 ± (30.1)	59.2 ± (29.2)	0.551*
Number of performed home exercise, mean (SD), times per week	1.4 ± (1.0)	1.3 ± (0.9)	0.687*
Minutes of walking, mean (SD), minutes per week	170.9 ± (119.4)	155.5 ± (108.6)	0.602*

SD, Standard deviation. P-values are for t-tests and p-values with markings (*) are from median based Mann-Whitney-U test

Number of participants with missing data: the number of performed home exercises, and walking minutes (non-sarcopenia, 11; sarcopenia, 3), fat percent (non-sarcopenia, 1)

**Table 2:** Means for Sarcopenia determinants of non-sarcopenia and sarcopenia groups and differences between groups

	Non-sarcopenia (*n* = 264)	Sarcopenia (*n* = 49)	*p*-value
Grip strength, mean (SD), kg	30.0 ± 10.4	16.0 ± 4.6	< 0.001
Knee extension strength, mean (SD), kg	38.5 ± 11.7	29.1 ± 10.4	< 0.001
Appendicular skeletal muscle mass, mean (SD), kg	19.7 ± 4.4	17.5 ± 3.7	< 0.001
Appendicular skeletal muscle mass index, mean (SD), kg/m^2^	7.0 ± 1.0	6.6 ± 1.0	0.005
Walking speed, mean (SD), m/s	1.3 ± 0.2	1.2 ± 0.2	< 0.001

SD, Standard deviation. P-values are for t-tests. Number of participants with missing data: knee extension strength (non-sarcopenia, 3), skeletal muscle mass (non-sarcopenia, 1)

The increase in grip strength in the sarcopenia group was on average 1.9 kg greater than in the non-sarcopenia group (interaction, β = 1.9, 95% CI 0.4–3.3, *p* =.014). There was no statistically significant difference in the change of knee extension strength, walking speed, or ASMI between the sarcopenia and non-sarcopenia groups. Still, both groups had statistically significant improvements in knee extension strength and habitual walking speed, while there were no changes in ASMI (Table 3).

**Table 3:** The level of and changes in outcomes from baseline to post-intervention in the Sarcopenia and non-sarcopenia groups from the generalized estimation equation models

		Pre	Post	Group effect		Time effect			Group×Time effect		
Group	N	EMM (SE)	EMM (SE)	Difference (95% CI)	p	Difference (95% CI)	p	Effect size	Difference (95% CI)	p	Effect size
Grip strength, kg											
SR	49	19.6 (0.6)	22.5 (0.8)	-11.6 (-12.9,-10.2)	< 0.001	2.9 (1.6, 4.3)	< 0.001	0.58	1.9 (0.4–3.3)	0.014	0.37
NSR	264	31.2 (0.4)	32.2 (0.5)	Reference		1.1 (0.5, 1.7)	< 0.001	0.21	Reference		
Knee extension strength, kg											
SR	49	33.4 (1.2)	37.2 (1.2)	-6.3 (-8.8, -3.8)	< 0.001	3.8 (2.5, 5.0)	< 0.001	0.85	-0.5 (-1.9, 0.9)	0.475	-0.10
NSR	261	39.7 (0.5)	44.0 (0.5)	Reference		4.3 (3.7, 4.9)	< 0.001	0.83	Reference		
Appendicular skeletal muscle mass index, kg/m^2^											
SR	49	6.9 (0.1)	6.8 (0.1)	-0.3 (-0.5, 0.0)	0.063	-0.1 (-0.2, 0.0)	0.261	-0.17	-0.1 (-0.2, 0.0)	0.249	-0.18
NSR	263	7.1 (0.0)	7.2 (0.0)	Reference		0.0 (-0.0, 0.1)	0.764	0.02	Reference		
Walking speed, m/s											
SR	49	1.2 (0.0)	1.3 (0.0)	-0.1 (-0.1, 0.0)	0.008	0.1 (0.0, 0.1)	0.004	0.41	-0.0 (-0.1, 0.0)	0.518	-0.11
NSR	263	1.3 (0.0)	1.4 (0.0)	Reference		0.1 (0.1,0.1)	< 0.001	0.57	Reference		

EMM, Estimated marginal mean; SE, standard error; CI, confidence interval; SR, sarcopenia group; NSR, non-sarcopenia group; ASMI, Appendicular skeletal muscle mass index

In the group effect analysis, the non-sarcopenia group was set as a reference group. In the Group×Time effect analysis, the non-sarcopenia group and the baseline measurement was set as reference. All models were adjusted for sex, age, resistance training adherence, and walking training adherence

In the pooled data, greater adherence to walking training was associated with a greater increase in knee extension strength in the sarcopenia group (β = 0.044, *p* =.005). In the non-sarcopenia group, greater adherence to resistance training was associated with a greater increase in grip strength (β = 0.026, *p* =.025), knee extension strength (β = 0.042, *p* =.004), and higher adherence to walking training was associated with a greater improvement in walking speed (β = 0.001, *p* =.029).

## Discussion

Compared to physical training following the physical activity recommendations for older adults, a combination of physical and cognitive training did not have additional effect on muscle strength, muscle mass, or walking speed in older adults with or without sarcopenia. Physical training increased knee extension strength and habitual walking speed similarly in participants with and without sarcopenia, while the increase in grip strength was greater in the sarcopenia group. No improvement or difference in ASMI changes was seen. This study provides evidence that multicomponent physical training according to physical activity guidelines can be as beneficial or even more beneficial for increasing strength in people with sarcopenia than in those without.

Although a combination of physical and cognitive training may provide small additive benefits for physical function, which is a key determinant of severe sarcopenia, compared to physical training alone, it may not provide additive benefits for muscle strength or muscle mass [15]. This is supported by our findings that cognitive training in addition to physical training did not show greater improvements in any component of sarcopenia in older adults with or without sarcopenia. Of note, the discrepancy with the findings of Gavelin et al. [15] may be explained by the fact that the present study sample consisted of relatively well-functioning older adults and did not include individuals with severe sarcopenia.

Although muscle mass did not increase in either study group, it was maintained over the 12-month period. Previous studies have shown that adults aged ∼75 years have an annual loss of muscle mass of 0.6 to 1% [20]. Chen et al. found that only interventions with moderate-to-vigorous intensity resistance training (greater than 70% of 1 repetition maximum) had an effect on ASMI in older adults [21]. In this study, the intensity of resistance training progressively increased above 70% of 1 repetition maximum, but the weekly frequency for gym-based resistance training was probably too low to increase muscle mass. To improve muscle mass in older adults, the frequency of weekly resistance training should be higher than in this study, about 2–3 times a week [22]. Although the intervention included home exercises with a resistance band, this may not provide sufficient loading to gain muscle mass, but to maintain it [23]. In the future, more intensive resistance training interventions may be recommended to increase muscle mass in older adults with sarcopenia. In addition, nutritional interventions, such as protein supplementation, may be recommended alongside resistance training to have greater improvement in muscle mass in older people [24, 25].

In our study grip strength, knee extension strength and walking speed increased after one year of multicomponent training. The effect size for improvements in knee extension strength was large in the sarcopenia and non-sarcopenia groups, whereas the effect sizes for grip strength and walking speed were from small to medium. As the intervention included more lower body exercise and walking, this explains why the intervention had a greater effect on lower limb muscle strength. In a recent meta-analysis exploring the effects of resistance training on muscle strength in the oldest adults (> 80 years), the effects of resistance training were seen in knee extension strength, but training had no effect on grip strength [26]. Grgic et al. [26] further discussed that in some cases, grip strength testing may provide limited insight into the effectiveness of resistance training programme, as training is often more focused on the lower body. According to our hypothesis, those with lower baseline strength values had greater improvements in strength compared to those with higher baseline values. This was only shown for grip strength and not for knee extension strength. This may be explained by the fact that there was a larger difference in grip strength (14 kg) compared to knee extension strength (9 kg) between the non-sarcopenia and sarcopenia groups.

The results of our study are consistent with a recent systematic review suggesting that combined resistance and aerobic training improves both muscle strength and physical performance in older adults with sarcopenia [27]. Our results are consistent as well with the recommendations for resistance training in the management and prevention of sarcopenia [28]. However, there is some debate about the frequency of the resistance training and whether once a week is sufficient [28]. Our study suggests that combined physical training including once a week instructed resistance training and home-based exercises is sufficient to increase muscle strength.

It is important to emphasize that physical training according to physical activity guidelines is effective to increase muscle strength and walking speed in sarcopenia group as shown in the present study. This is noteworthy since physical activity guidelines are well promoted in general population and in different health care settings as well. Currently, the implementation of physical activity guidelines among older people is still low. In the most recent report among the Finnish population, about one third or less of adults aged 65 years and over, achieved the physical activity target [29], while the proportion of people meeting the guidelines in the Europe is less than 10% [30]. Therefore, we should focus on improving people’s adherence to physical activity guidelines aiming to prevent and manage sarcopenia more effectively. We need multiple strategies to increase physical activity of older people, such as increasing social and environmental support, improving access to physical activity facilities, organising group exercise, and supporting health-care systems to implement exercise-based programmes in older adults [31], as well as promoting physical activity through applications of behaviour change theories [32].

As successful implementation of physical activity guidelines has positive effects on sarcopenia determinants, such as muscle strength and walking speed, it may also help to maintain the physical function in older people [33]. Good physical function promotes functional independence and health-related quality of life [34, 35]. In addition, higher levels of muscle strength and good physical performance are important factors in preventing falls [36] and decreasing healthcare costs [37]. From a physiological point of view, maintaining muscle mass plays an important role in the prevention of age-related diseases, such as insulin resistance, chronic inflammation or cardiovascular diseases [38, 39].

To our best knowledge, only a few studies have investigated the effects of long-term combined resistance, and aerobic training on sarcopenia. In the latest meta-analysis, which included a total of 48 trials that studied the effects of different training programs on sarcopenia, the average duration of the interventions was 17.4 weeks, and two trials lasted up to 48 weeks [40]. The results showed that resistance training was best for improving grip strength and muscle mass, while non-resistance training was best for increasing walking speed [40]. Recently conducted SPRINTT -study investigated whether 36-months of moderate-intensity PA training, including aerobic, strength, balance, and flexibility exercises, had an effect on grip strength and muscle mass in frail people compared with a group that received education about healthy ageing. In the SPRINTT -study grip strength and lean mass decreased during the 36-month period, but the decrease was less in the intervention group than in the control group [41].

In this study adherence to the 12-month training was relatively high and the attrition rate was low, which was also seen in the SPRINTT study [41]. Important finding was that adherence to training was similar between the sarcopenia and non-sarcopenia groups. In this study, there were several factors that have previously been identified as playing a key role in helping people to adhere to training, including a multidisciplinary training programme and supervision in group-based sessions that provide social support, relatedness and feedback [42]. In addition, the use of air-pressure machines equipped with the HUR SmartCard system that remembers the user’s settings and resistance allowed for easy and enjoyable resistance training and monitoring, that could have an impact to adherence. In addition, the adherence to supervised training sessions was associated with the changes in muscle strength and walking speed. This highlights, that during interventions, maintaining weekly training routines are mandatory for optimal gains.

## Strengths and limitations

Physical training protocol was designed according to ACSM physical activity guidelines [17] and was adapted from our previous study [43] and the LIFE study [44], including supervised group exercise sessions. Supervised sessions enabled the control of progression and adherence to training according to physical activity guidelines. In addition, intervention was long, and commitment to 12-month training was generally high and attrition rate was relatively low.

As a limitation, this was an exploratory study of the original PASSWORD -study, the primary objective which was to investigate effects on maximal walking speed and executive function. Because of the explorative nature of the study, there were no power calculations performed for the analysis, and no passive or usual care control group were involved. In addition, relatively healthy people participated to the present study and therefore the effectiveness of intervention could be greater when studying diagnosed sarcopenia individuals. Finally, the total number of participants in the sarcopenia group was relatively low compared to the without sarcopenia group.

## Conclusion

A yearlong combined physical training including components from physical activity recommendations seem to be effective in increasing maximal grip, knee extension strength, and habitual walking speed in older adults with or without sarcopenia. This evidence supports that moderate intensity, and long-term combined physical training may be effective in preventing sarcopenia. More intensive resistance training may be needed to increase muscle mass in older adults. Cognitive training in addition to physical training had no beneficial effects on strength, muscle mass or walking speed.

## Electronic supplementary material

Below is the link to the electronic supplementary material.

Supplementary Material 1

## Data Availability

Datasets used during the current study are available from the author (SS) on reasonable request.
