# Supplementary material for: Effects of 12-month physical and cognitive training on sarcopenia determinants in older adults: a subgroup analysis of a randomised clinical trial
Source: Aging Clin Exp Res. 2025 Feb 6;37(1):36. doi: 10.1007/s40520-025-02935-7 (PMC11802700; doi:10.1007/s40520-025-02935-7)
Supplement: Supplementary file 1 — Supplementary Material 1 [file 40520_2025_2935_MOESM1_ESM.docx]

Supplementary table 1. The level of and changes in outcomes from baseline to post-intervention in the PTCT and PT groups from the generalized estimation equation models in participants with sarcopenia.

| Sarcopenia |  | Pre | Post | Group effect |  | Time effect | | Group×Time effect | |
| --- | --- | --- | --- | --- | --- | --- | --- | --- | --- |
| Group | N | EMM (SE) | EMM (SE) | Difference (95% CI) | p | Difference (95% CI) | p | Difference (95% CI) | p |
| Grip strength, kg | |  |  |  |  |  |  |  |  |
| PTCT | 28 | 17.3 (0.4) | 19.9 (0.8) | -1.1 (-2.3, 0.1) | 0.080 | 2.6 (0.9, 4.3) | 0.003 | -0.3 (-0.3, 2.4) | 0.817 |
| PT | 21 | 18.4 (0.4) | 21.3 (1.0) | Reference |  | 2.9 (0.8, 5.0) | 0.006 | Reference |  |
| Knee extension strength, kg | | |  |  |  |  |  |  |  |
| PTCT | 28 | 31.7 (1.7) | 34.5 (1.7) | 0.0 (-4.1, 4,2) | 0.982 | 2.8 (1.4, 4.2) | <0.001 | -2.3 (-4.8, 0.2) | 0.075 |
| PT | 21 | 31.6 (1.7) | 36.7 (1.8) | Reference |  | 5.1 (3.0, 7.2) | <0.001 | Reference |  |
| ASMI, kg/m^2^ | | |  |  |  |  |  |  |  |
| PTCT | 28 | 6.7 (0.1) | 6.7 (0.1) | -0.2 (-0.8, 0.4) | 0.478 | -0.1 (-0.2, 0.1) | 0.508 | 0.0 (-0.2, 0.2) | 0.955 |
| PT | 21 | 6.9 (0.3) | 6.9 (0.3) | Reference |  | -0.1 (-0.2, 0.1) | 0.317 | Reference |  |
| Walking speed, m/s | |  |  |  |  |  |  |  |  |
| PTCT | 28 | 1.2 (0.0) | 1.3 (0.0) | 0.1 (-0.0, 0.2) | 0.220 | 0.1 (0.0, 0.2) | 0.037 | -0.1 (-1.4, 0.4) | 0.282 |
| PT | 21 | 1.2 (0.0) | 1.3 (0.0) | Reference |  | 0.1 (0.0, 0.2) | 0.025 | Reference |  |

Supplementary table 2. The level of and changes in outcomes from baseline to post-intervention in the PTCT and PT groups from the generalized estimation equation models in participants without sarcopenia.

| Non-sarcopenia |  | Pre | Post | Group effect |  | Time effect | | Group×Time effect | |
| --- | --- | --- | --- | --- | --- | --- | --- | --- | --- |
| Group | N | EMM (SE) | EMM (SE) | Difference (95% CI) | p | Difference (95% CI) | p | Difference (95% CI) | p |
| Grip strength, kg | |  |  |  |  |  |  |  |  |
| PTCT | 121 | 31.0 (0.5) | 32.5 (0.6) | -0.71 (-2.3, 0.8) | 0.361 | 1.5 (0.7, 2.4) | <0.001 | 0.98 (-0.2, 2.2) | 0.110 |
| PT | 138 | 31.7 (0.6) | 32.3 (0.6) | Reference |  | 0.6 (-0.3, 1.4) | 0.177 | Reference |  |
| Knee extension strength, kg | | |  |  |  |  |  |  |  |
| PTCT | 128 | 39.8 (0.7) | 44.0 (0.7) | -0.3 (-2.2, 1.5) | 0.695 | 3.8 (1.9, 5.7) | <0.001 | -0.2 (-1.5, 1.0) | 0.696 |
| PT | 138 | 40.2 (0.7) | 44.5 (0.6) | Reference |  | 4.4 (3.5, 5.3) | <0.001 | Reference |  |
| ASMI, kg/m^2^ | | |  |  |  |  |  |  |  |
| PTCT | 121 | 7.2 (0.1) | 7.2 (0.1) | 0.0 (-0.1, 0.2) | 0.599 | 0.0 (-0.1, 0,2) | 0.670 | -0.0 (-0.1, 0.1) | 0.508 |
| PT | 138 | 7.1 (0.1) | 7.2 (0.1) | Reference |  | 0.0 (-0.0, 0.1) | 0.475 | Reference |  |
| Walking speed, m/s | |  |  |  |  |  |  |  |  |
| PTCT | 122 | 1.3 (0.0) | 1.4 (0.0) | 0.0 (-0.0, 0.1) | 0.143 | 0.1 (-0.0, 0.1) | <0.001 | -0.0 (-0.0, 0.0) | 0.072 |
| PT | 138 | 1.3 (0.0) | 1.4 (0.0) | Reference |  | 0.1 (0.1, 0.1) | <0.001 | Reference |  |
